# Consensus goals and standards for specialist cough clinics: the NEUROCOUGH international Delphi study

**DOI:** 10.1183/23120541.00618-2023

**Published:** 2023-11-20

**Authors:** Woo-Jung Song, Lieven Dupont, Surinder S. Birring, Kian Fan Chung, Marta Dąbrowska, Peter Dicpinigaitis, Christian Domingo Ribas, Giovanni Fontana, Peter G. Gibson, Laurent Guilleminault, James H. Hull, Marco Idzko, Peter Kardos, Hyun Jung Kim, Kefang Lai, Federico Lavorini, Eva Millqvist, Alyn H. Morice, Akio Niimi, Sean M. Parker, Imran Satia, Jaclyn A. Smith, Jan Willem van den Berg, Lorcan P. McGarvey

**Affiliations:** 1 Department of Allergy and Clinical Immunology, Asan Medical Center, University of Ulsan College of Medicine, Seoul, Korea; 2 Department of Respiratory Diseases, University Hospital Leuven, Katholieke Universiteit Leuven, Leuven, Belgium; 3 Centre for Human and Applied Physiological Sciences, School of Basic and Medical Biosciences, King's College London, London, UK; 4 Experimental Studies Unit, National Heart and Lung Institute, Imperial College London, London, UK; 5 Department of Internal Medicine, Pulmonary Diseases and Allergy, Medical University of Warsaw, Warsaw, Poland; 6 Albert Einstein College of Medicine and Montefiore Medical Center Bronx, Bronx, NY, USA; 7 Servicio de Neumología, Hospital Parc Taulí, Sabadell, Autonomous University of Barcelona (UAB), Barcelona, Spain; 8 Department of Experimental and Clinical Medicine, University of Florence, Florence, Italy; 9 School of Medicine and Public Health, University of Newcastle, Newcastle, NSW, Australia; 10 Service de Pneumologie-Allergologie, Pôle des Voies Respiratoires, Hôpital Larrey and Center for Pathophysiology Toulouse Purpan, INSERM U1043, CNRS UMR 5282, Toulouse III University, Toulouse, France; 11 Royal Brompton Hospital, Guy's and St Thomas’ NHS Trust, London, UK; 12 Department of Pneumology, University Hospital Vienna AKH, Medical University of Vienna, Vienna, Austria; 13 Centre of Allergy, Respiratory and Sleep Medicine, Maingau Clinic of the Red Cross, Frankfurt am Main, Germany; 14 Institute for Evidence-Based Medicine, Cochrane Korea, Department of Preventive Medicine, Korea University College of Medicine, Seoul, Korea; 15 The First Affiliated Hospital of Guangzhou Medical University, National Center of Respiratory Medicine, State Key Laboratory of Respiratory Disease, Guangzhou Institute of Respiratory Health, Guangzhou, China; 16 Department of Allergology, Institution of Internal Medicine, The Sahlgrenska Academy at the University of Gothenburg, Gothenburg, Sweden; 17 Centre for Clinical Science, Respiratory Medicine, Hull York Medical School, University of Hull, Castle Hill Hospital, Cottingham, UK; 18 School of Medical Sciences, Nagoya City University, Nagoya, Japan; 19 North Tyneside General Hospital, North Shields, UK; 20 Department of Medicine, McMaster University and Firestone Institute for Respiratory Health, St Joseph's Healthcare, Hamilton, Canada; 21 Division of Immunology, Immunity to Infection and Respiratory Medicine, University of Manchester and Manchester University NHS Trust, Manchester, UK; 22 Department of Pulmonology, Isala Hospital, Zwolle, The Netherlands; 23 Wellcome-Wolfson Institute for Experimental Medicine, School of Medicine, Dentistry and Biomedical Sciences, Queen's University Belfast, Belfast, UK

## Abstract

**Background:**

Current guidelines on the management of chronic cough do not provide recommendations for the operation of specialist cough clinics. The objective of the present study was to develop expert consensus on goals and standard procedures for specialist cough clinics.

**Methods:**

We undertook a modified Delphi process, whereby initial statements proposed by experts were categorised and presented back to panellists over two ranking rounds using an 11-point Likert scale to identify consensus.

**Results:**

An international panel of 57 experts from 19 countries participated, with consensus reached on 15 out of 16 statements, covering the aims, roles and standard procedures of specialist cough clinics. Panellists agreed that specialist cough clinics offer optimal care for patients with chronic cough. They also agreed that history taking should enquire as to cough triggers, cough severity rating scales should be routinely used, and a minimum of chest radiography, spirometry and measurements of type 2 inflammatory markers should be undertaken in newly referred patients. The importance of specialist cough clinics in promoting clinical research and cough specialty training was acknowledged. Variability in healthcare resources and clinical needs between geographical regions was noted.

**Conclusions:**

The Delphi exercise provides a platform and guidance for both established cough clinics and those in planning stages.

## Tweetable abstract

**A Delphi exercise undertaken to provide expert consensus on goals and standard procedures for establishing and delivering a specialist cough clinic service** https://bit.ly/3Qopt6z

## Introduction

Cough is one of the most common reasons why patients seek medical attention [1, 2]. Chronic cough, usually defined as a cough persisting for more than 8 weeks, is globally prevalent and associated with impaired quality of life [3–5]. It contributes to social isolation, depression, work productivity impairment and considerable healthcare burden [6–12].

Despite recent advances in our understanding of cough neuro-pathophysiology and broad consensus on how best to evaluate and manage patients troubled with chronic cough [13–22], the effective treatment remains a significant unmet clinical need. Although chronic cough is frequently associated with medical conditions such as asthma, postnasal drip or gastro-oesophageal reflux disease, the cough may remain unexplained or persist for several years or decades despite meticulous evaluation and treatment in some patients [23–25]. Such patients are desperate for a diagnosis and treatment but often find themselves helpless in their healthcare journey [26–29]. Typically they report difficulty in locating cough specialists [30, 31] and the impact of chronic cough is not properly understood by families or physicians because it is often considered as a symptom of other diseases and fairly trivial [4, 28, 30]. Some patients may end up seeing many specialists to seek a diagnosis and are often overly or inadequately investigated [9, 28, 29, 32, 33].

Poor implementation of existing cough guidelines is also a challenge [28, 34]. In one recent community population survey, chest imaging, which is routinely recommended, was only performed in about 40% of patients with chronic cough, while bronchoscopy (8.1–14.9%) and gastrointestinal testing (8.9–12.4%) were undertaken more frequently than recommended [28]. Further discordance from recommendations has been reported elsewhere, with over-requesting of chest imaging and an over-prescription of antibiotics [35]. Further, a recent online survey of Canadian physicians (general practitioners and hospital specialist) revealed many are not using current cough management guidelines [34]. While consensus has been sought on how chronic cough patients should be assessed in primary care prior to specialist referral, there is clearly a need to improve the quality of care provided to this patient group [36].

While there has been a breakthrough in the pharmacological management of refractory chronic cough [37], we believe that specialist cough clinics should play core roles in addressing unmet clinical needs and advancing the field. The NEw Understanding in the tReatment Of COUGH (NEUROCOUGH) Clinical Research Collaboration (CRC) endorsed by the European Respiratory Society is a pan-European multicentre network established to improve the management of chronic cough [38]. A specific aim was to establish consensus on the goals and standard procedures for specialist cough clinics. To achieve this, we conducted an online Delphi survey of international cough experts and clinicians seeking consensus criteria that will lead to the delivery of optimal care, thereby improving the quality of care for patients with chronic cough.

## Methods

### Study design

We used a modified Delphi method to develop consensus goals and standard procedures in specialist cough clinics. A working group of cough experts in the NEUROCOUGH CRC was convened to brainstorm and draft statements and survey items. The items and statements were drafted based on recent cough clinic surveys, literature and iterative discussion among the expert working group (supplementary table E1). The statements were not meant to be hierarchical or all-inclusive but were chosen to cover the major concerns practicing clinicians have in relation to cough clinics.

### Panellists

E-mail invitations were sent to 74 clinicians (from 19 countries) actively involved in the management of patients with chronic cough. They comprised NEUROCOUGH CRC National Leads, clinicians on the International Advisory Board or other clinicians recommended by NEUROCOUGH CRC members. Our aim was to include panellists from many countries and diverse healthcare settings.

### Delphi process

Two rounds of online surveys were conducted over 12 months, using SurveyMonkey online software (www.surveymonkey.co.uk). Panellists were given 4 weeks to reply to each survey round. If responses were not received, an e-mail reminder was sent to individual participants.

Each statement was ranked using an 11-point Likert scale (0–10), ranging from “strongly disagree” (0) to “neither agree or disagree” (5) and “strongly agree” (10). The responses were then categorised as negative (0–2), neutral (3–7) and positive (8–10). For statements relating to the rating of item importance, such as routine diagnostic tests, cough assessment tools or cough clinic quality indicators, a 5-point Likert scale (0–4) was utilised, ranging from “not important at all” (0) to “neutral” (2) and “very important” (4). Statements were refined over two Delphi rounds. After Round 1, the statements and items were adapted or reworded to reflect the comments and suggestions from the panellists. Round 1 aimed to rank importance or agreement on each item and to explore the level of preliminary consensus. In Round 2, we aimed to achieve a consensus: each participant received an individualised survey, comprising all statements marked with revisions presented with the whole panel's group response. Participants were then asked to reconsider their responses in light of the panel responses for a final time.

Our predetermined criteria for deciding when consensus was reached were: all votes required 75.0% participation of those eligible to vote; and consensus was achieved if “1) >60.0% of respondents were positive (8–10) or negative (0–2)” AND “2) the proportion of opponent group (0–2 or 8–10, respectively) was <20.0%”. Consensus was defined as “very high” at >90%.

### Analysis

Descriptive analyses were conducted with Stata version 15.1 (StataCorp, College Station, TX, USA). Graphs were drawn using Prism version 9.5 (GraphPad, La Jolla, CA, USA).

## Results

**FIGURE 1:**
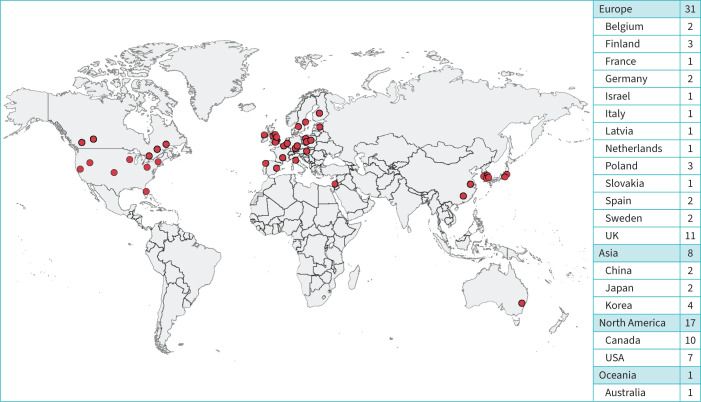
Geographical distribution of clinics among 57 panellists from 19 countries.

Among 74 cough experts and clinicians invited, 57 subjects (77.0%) from 19 countries agreed to participate as the panel (supplementary table E2). The geographic distribution of panellists is presented in figure 1; 79.6% were practicing in tertiary care and 22.4% in secondary care.

**FIGURE 2:**
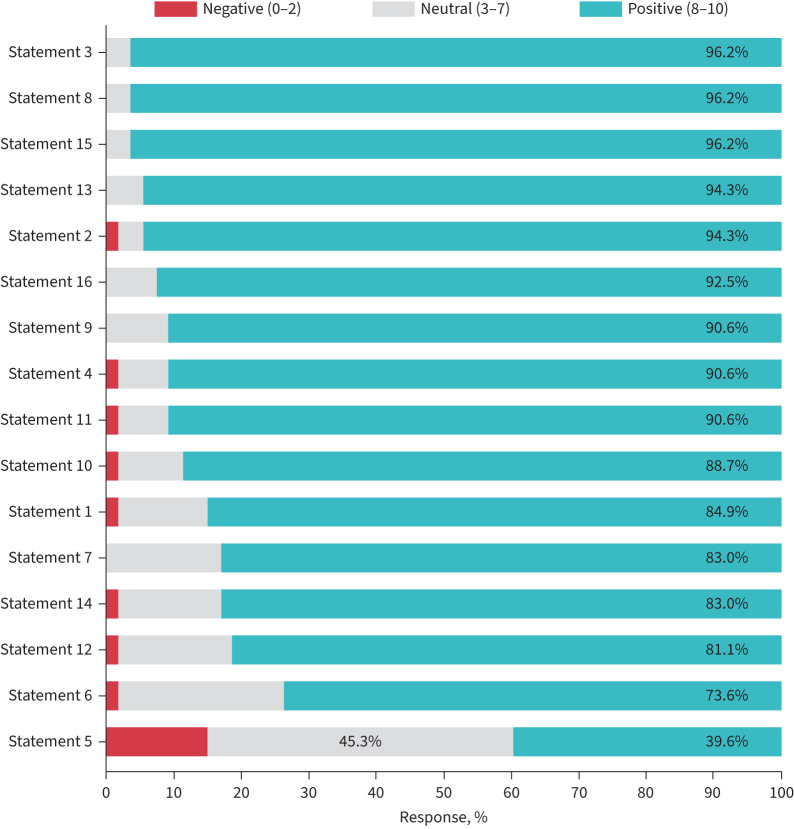
Panellist responses to statements. Each statement was ranked using an 11-point Likert scale (0–10), ranging from “strongly disagree” (0) to “neither agree nor disagree” (5) and “strongly agree” (10). Responses were categorised as negative (0–2), neutral (3–7) and positive (8–10). Statements are listed from top (most agreement) to bottom (least agreement).

The panel reached consensus on 15 out of 16 statements, covering the aims, roles and standard procedures of specialist cough clinics. The final consensus statements with level of consensus are presented in table 1 and figure 2, and outstanding remarks for each statement provided in the following text.

**TABLE 1:** Statements that achieved consensus

**Statement**		**Positive response (8–10), %**
**1**	Specialist cough clinics should be established to provide the optimal care for patients with chronic cough and refractory cough.	84.9
**2**	Aims of specialist cough clinics should be to improve patient outcomes, optimise investigations and treatment, reduce burden of disease, and advance clinical research through patient registries and interventional clinical trials.	94.3
**3**	Specialist cough clinics should be supervised by clinicians with expertise in cough management.	96.2
**4**	Specialist cough clinics should provide evaluation and management of chronic cough patients guided by the agreed national and/or international consensus. Such standardised management should also be encouraged in general respiratory, allergy or ENT clinics responsible for the care of patients with chronic cough.	90.6
**6**	Cough should be routinely assessed at baseline and follow-up, using a rating scale for cough severity (such as 0–10 score, modified Borg scale, a 0–100 visual analogue scale or an appropriate alternative). Cough-specific quality of life should also be a part of the assessment at specialist cough clinics, particularly for research purposes.	73.6
**7**	Cough triggers and cough complications should be a part of routine history taking, preferably by means of validated measurement tools.	83.0
**8**	In every patient newly referred with chronic cough, a minimum panel of routine tests should be reviewed, or undertaken if not already performed. The minimum panel of tests are 1) chest X-ray, 2) spirometry (with bronchodilator testing if indicated) and 3) a type 2 inflammatory marker (such as blood eosinophils, fractional exhaled nitric oxide (*F*_ENO_) or sputum eosinophils).	96.2
**9**	Decision to commence opiates (as anti-tussive therapy) should be made by clinicians with expertise in cough management.	90.6
**10**	Decision to commence current neuromodulators (such as gabapentin or amitriptyline, as anti-tussive therapy) should be made by clinicians with expertise in cough management.	88.7
**11**	Cough control therapy, or speech language and pathology therapy, should be available in specialist cough clinics.	90.6
**12**	In specialist cough clinics, multidisciplinary team meetings should take place to discuss complex cough patients.	81.1
**13**	Specialist cough clinics should offer opportunities to patients to participate in clinical research or trials of novel cough therapies.	94.3
**14**	Specialist cough clinics should participate in local and international audit on an ongoing basis with the aim of providing a high-quality cough service.	83.0
**15**	Cough evaluation and management should be integrated into the post-graduate specialty (*e.g.* respiratory, allergy or ENT) training curriculum.	96.2
**16**	Specialty trainees/fellows (*e.g.* respiratory, allergy or ENT) should be required to undertake a period of training/participate in clinics which regularly receive referrals for chronic cough.	92.5

ENT: ear, nose and throat.

### Statement 1

*Specialist cough clinics should be established to provide the optimal care for patients with chronic cough and refractory cough.*

The majority of panellists (84.9%) agreed.

Remark: The specific operations of cough clinics may depend on geography, healthcare resources, referral system or clinical needs in each region. Specialist clinics should be focused on the care of patients with refractory chronic cough. Some panellists expressed concerns that it may not be feasible or practical to set up specialist cough clinics in sparsely populated, low- and middle-income or small countries; however, virtual clinics might be an alternative.

### Statement 2

*Aims of specialist cough clinics should be to improve patient outcomes, optimise investigations and treatment, reduce burden of disease, and advance clinical research through patient registries and interventional clinical trials* *.*

Consensus was very high (94.3% positive).

Remark: As healthcare resources may differ between countries, they should be taken into consideration when implementing the statement.

### Statement 3

*Specialist cough clinics should be supervised by clinicians with expertise in cough management.*

Consensus was very high (96.2% positive).

Remark: Healthcare administrative rules may differ by country and they need to be taken into consideration when implementing the statement. For example, clinics should be led by doctors in some countries, whereas nurse-led clinics supported by a telemedicine service with a cough specialist doctor may be an option in other countries.

### Statement 4

*Specialist cough clinics should provide evaluation and management of chronic cough patients guided by the agreed national and/or international consensus. Such standardised management should also be encouraged in general respiratory, allergy or ENT clinics responsible for the care of patients with chronic cough* *.*

The majority of panellists (90.6%) agreed. After Round 1, a sentence “*Such standardised management should also be encouraged in general respiratory, allergy or ENT clinics responsible for the care of patients with chronic cough.*” was added.

Remark: There were comments from some panellists on 1) low level of certainty of current clinical evidence, 2) discrepancy existing even among clinicians with expertise in patient management and 3) potential risk of prohibiting innovation or new approaches. The consensus indicates general principles in management of patients with chronic cough which should be individualised according to the patient's traits, local administrative rules and healthcare resource availability. Meanwhile, best practice for cough is still evolving; specialist cough clinics have a major role in research and should consider novel strategies outside existing guidelines in the context of ethically approved clinical studies.

### Statement 5

*Specialist cough clinics should be established in every secondary or tertiary respiratory, allergy or ENT care facility.*

Consensus was not achieved because only 39.6% of responses were positive; 15.1% were negative and 45.3% were neutral. Some panellists pointed out limited feasibility and practicality of establishing dedicated cough clinics in every secondary or tertiary respiratory or allergy care facility, while agreeing with the concept. It was commented that geography, healthcare resources or clinical needs may be different between countries.

### Statement 6

*Cough should be routinely assessed at baseline and follow-up, using a rating scale for cough severity (such as 0–10 severity score* *, modified Borg scale, a 0–100 visual analogue scale or an appropriate alternative). Cough-specific quality of life should also be a part of the assessment at specialist cough clinics, particularly for research purposes.*

Consensus was achieved (73.6% positive). This statement was refined based on the responses in Round 1 with inclusion of specific cough assessment tools.

Remark: Two items, *i.e.* cough severity numerical rating scale and cough-specific quality of life, were endorsed by the respondents as they were considered to be important or very important by more than 70.0% of panellists (table 2). However, cough-specific QoL tools may be more appropriate for research purposes and mandating different questionnaires may pose a burden to patients and clinicians.

**TABLE 2:** Rating^#^ of item importance as a routine assessment tool in cough clinics^¶^

	**Rating**				
	**0**	**1**	**2**	**3**	**4**
**Cough severity numerical rating scale (*e.g.* 0–10, modified Borg scale or 0–100 visual analogue scale)**	0	1.9	3.8	22.6	71.7
**Cough-specific impact or QoL (*e.g.* LCQ or CQLQ)**	0	11.3	15.1	37.7	35.9
**Airway reflux questionnaire (*e.g.* HARQ)**	9.4	17.0	28.3	32.1	13.2
**Cough severity diary**	5.7	11.3	39.6	36.9	7.5
**Cough frequency (ambulatory cough monitoring)**	11.3	18.9	41.5	17.0	11.3
**General health QoL (*e.g.* EuroQoL or SF-36)**	17.0	30.2	43.4	5.6	3.8

Data are presented as response %. QoL: quality of life; LCQ: Leicester Cough Questionnaire; CQLQ: Cough-specific Quality of Life Questionnaire; HARQ: Hull Airway Reflux Questionnaire; SF-36: 36-item Short-Form Health Survey. ^#^: 5-point Likert scale (0–4), ranging from “not important at all” (0) to “neutral” (2) and “very important” (4); ^¶^: original question: Please rate the importance of each item as a routine assessment tool in cough clinics.

### Statement 7

*Cough triggers and cough complications should be a part of routine history taking, preferably by means of validated measurement tools.*

Consensus was agreed (83.0% positive). This statement was added in Round 2, on the basis of Round 1 responses regarding additional items in cough assessment (table 3).

**TABLE 3:** Rating^#^ of item importance as additional aspect of cough that should be routinely measured in patients referred to cough clinics^¶^

	**Rating**				
	**0**	**1**	**2**	**3**	**4**
**Cough complications**	0	0	5.7	24.5	69.8
**Cough triggers**	0	1.9	5.7	24.5	67.9
**Throat sensations**	0	1.9	13.2	52.8	32.1
**Urge to cough**	1.9	3.8	20.8	41.5	32.1
**Subjective cough frequency**	1.9	5.7	15.1	47.2	30.2

Data are presented as response %. ^#^: 5-point Likert scale (0–4), ranging from “not important at all” (0) to “neutral” (2) and “very important” (4); ^¶^: original question: Please rate the importance of each item as additional aspect of cough that should be routinely measured in patients referred to cough clinics (we recognise that specific assessment tools may not currently exist).

Remark: More than 90.0% of panellists considered 1) cough complications and 2) cough triggers (table 3). However, at present, there is a paucity of validated tools for evaluation of either.

### Statement 8

*In every patient newly referred with chronic cough, a minimum panel of routine tests should be reviewed, or undertaken if not already performed. The minimum panel of tests are 1) chest X-ray, 2) spirometry (with bronchodilator testing if indicated) and 3) a type 2 inflammatory marker (such as blood eosinophils, fractional exhaled nitric oxide (*F*_ENO_) or sputum eosinophils).*

Consensus was very high (96.2% positive). The statement was refined with the specific minimum panel of routine diagnostic tests to be reviewed or undertaken (chest radiography (X-ray), spirometry and type 2 inflammatory markers), on the basis of Round 1 responses to item importance rating on routine diagnostic tests (table 4).

**TABLE 4:** Rating^#^ of item importance as a routine diagnostic test to be reviewed (or undertaken if not already performed) in every newly referred patient with chronic cough^¶^

	**Rating**				
	**0**	**1**	**2**	**3**	**4**
**Chest X-ray**	0	0	0	11.3	88.7
**Spirometry**	0	0	0	20.8	79.2
***F*_ENO_**	0	0	13.2	34.0	52.8
**Blood eosinophils**	0	3.8	17.0	28.3	50.9
**Reversibility test**	3.8	1.9	15.1	24.5	54.7
**Allergy skin test (or serum specific IgE test)**	3.8	13.2	22.6	30.2	30.2
**Methacholine challenge test**	7.5	13.2	17.0	32.1	30.2
**Sinus imaging**	13.2	11.3	28.3	26.4	20.8
**Sputum eosinophils**	9.4	24.5	20.8	24.5	20.8
**Nasal endoscopy**	9.4	18.9	32.1	24.5	15.1
**Laryngoscopy**	13.2	17.0	24.5	30.2	15.1
**24-h oesophageal pH test**	9.4	22.6	32.1	24.5	11.3
**Mannitol challenge test**	13.2	20.8	39.6	20.8	5.7
**High-resolution oesophageal manometry**	15.1	18.9	39.6	18.9	7.6
**Cough challenge test**	15.1	32.1	20.7	26.4	5.7
**GI endoscopy**	22.6	20.8	37.7	11.3	7.6
**Bronchoscopy**	20.8	24.5	34.0	20.8	0

Data are presented as response %. *F*_ENO_: fractional exhaled nitric oxide; GI: gastrointestinal. ^#^: 5-point Likert scale (0–4), ranging from “not important at all” (0) to “neutral” (2) and “very important” (4); ^¶^: original question: Please rate the importance of each item as a routine test to review (or undertake if not already performed) in every newly referred patient with chronic cough.

Remark: Three items were endorsed by the majority of respondents (rated important or very important by more than 80.0%): chest radiography, spirometry and type 2 inflammatory markers, such as *F*_ENO_ or blood eosinophils, and thus were chosen as the minimum panel of testing. Bronchial challenge tests and upper airway and gastrointestinal investigations should not be offered routinely but may be considered based on clinical judgement.

### Statement 9

*Decision to commence opiates (as anti-tussive therapy) should* *be made by clinicians with expertise in cough management.*

Consensus was very high (90.6% positive).

Remark: Local administrative rules should be taken into consideration when implementing the statement. Some panellists emphasised the use of opiates should be limited to specialist cough clinics, although this may overwhelm cough clinics, or in their absence, may limit such treatment options.

### Statement 10

*Decision to commence current neuromodulators (such as gabapentin or amitriptyline, as anti-tussive therapy) should* *be made by clinicians with expertise in cough management.*

Consensus was high (88.7% positive).

Remark: Local administrative rules should be taken into consideration when implementing the statement. One panellist commented on the needs for developing a consensus on the dosing, duration and tapering of the neuromodulators.

### Statement 11

*Cough control therapy, or speech and language pathology therapy, should be available in specialist cough clinics.*

A very high level of consensus was reached (90.6% positive).

Remark: Many agreed that cough control therapy (or speech and language pathology therapy) is an important part of the management. However, it is also recognised that the service is not accessible in many countries and regions. The pool of individuals with expertise in cough control therapy is currently lacking and should be increased. Experienced practitioners or physiotherapists should undertake the intervention.

### Statement 12

*In specialist cough clinics, multidisciplinary team meetings should take place to discuss complex cough patients.*

Consensus was achieved (81.1% positive).

Remark: Structures of cough clinics and healthcare systems may differ by region, and thus they should be taken into consideration when implementing the statement. Some panellists expressed concern about the feasibility and resource issues. The multidisciplinary team meetings (which may comprise speech and language therapy, gastroenterology, ENT and/or physiotherapy) may be useful to discuss complex cough problems but are not practical or possible in every clinical setting.

### Statement 13

*Specialist cough clinics should offer opportunities to patients to participate in clinical research or trials of novel cough therapies.*

Consensus was very high (94.3% positive).

Remark: It is the role of specialist clinics to advance knowledge and facilitate clinical trials with novel anti-tussive drugs.

### Statement 14

*Specialist cough clinics should participate in local and international audit on an ongoing basis with the aim of providing a high-quality cough service.*

Consensus was high (83.0% positive).

Remark: Several items were considered important or very important as quality indicators by more than 80.0% of panellists (table 5), such as the presence of clinicians with expertise in cough management, quantification of baseline cough severity/impact and treatment responses using established tools, diagnostic test accessibility or participation in clinical trials. These may serve as quality indicators. However, research is recommended to assess the outcomes of current cough clinical services and to optimise the care through audits.

**TABLE 5:** Rating^#^ of item importance as a quality indicator for cough clinical service^¶^

	**Rating**				
	**0**	**1**	**2**	**3**	**4**
**Presence of clinicians with expertise in cough management**	0	0	0	3.8	96.2
**Accessibility to spirometry**	0	0	0	7.6	92.4
**Accessibility to chest X-ray**	0	0	1.9	5.7	92.4
**Quantification of treatment response at follow-up consultation using established tools**	0	1.9	0	28.3	69.8
**Quantification of baseline cough severity or impact using established tools**	0	0	3.8	35.8	60.4
**Accessibility to cough control therapy (or speech language and pathology therapy)**	0	0	7.6	35.8	56.6
**Accessibility to *F*_ENO_**	0	0	17.0	26.4	56.6
**Accessibility to blood eosinophils**	1.9	3.8	9.4	22.6	62.3
**Accessibility to chest CT scan**	3.8	1.9	5.7	28.3	60.4
**Participation in clinical trials for novel cough therapies**	0	0	9.4	43.4	47.2
**Adherence to the agreed procedures defined by national and/or international consensus in patient management**	0	1.9	11.3	37.7	49.1
**Multidisciplinary team meeting**	0	5.7	18.9	34.0	41.5
**Accessibility to allergy skin test (or serum specific IgE test)**	3.8	3.8	18.9	32.1	41.5
**Accessibility to methacholine challenge test**	5.7	9.4	9.4	34.0	41.5
**Accessibility to sinus imaging**	7.6	7.6	15.1	22.6	47.2
**Accessibility to laryngoscopy**	1.9	5.7	24.5	32.1	35.9
**Accessibility to nasal endoscopy**	1.9	11.3	24.5	26.4	35.9
**Accessibility to bronchoscopy**	7.6	7.6	11.3	41.5	32.1
**Accessibility to 24-h oesophageal pH**	3.8	9.4	15.1	49.1	22.6
**Accessibility to high-resolution oesophageal manometry**	7.6	7.6	22.6	47.2	15.1
**Accessibility to GI endoscopy**	11.3	15.1	22.6	34.0	17.0
**Accessibility to sputum eosinophils**	15.1	15.1	18.9	28.3	22.6
**Accessibility to cough challenge test**	15.1	15.1	26.4	32.1	11.3
**Accessibility to mannitol challenge test**	9.4	24.5	28.3	35.9	1.9

Data are presented as response %. *F*_ENO_: fractional exhaled nitric oxide; CT: computed tomography; GI: gastrointestinal. ^#^: 5-point Likert scale (0–4), ranging from “not important at all” (0) to “neutral” (2) and “very important” (4); ^¶^: original question: Please rate the importance of each item as a quality indicator for cough clinical service. Accessibility to a certain test or therapy means that it can be done at the site or by referral.

### Statement 15

*Cough evaluation and management should be integrated into the post-graduate specialty (*e.g. *respiratory, allergy or ENT) training curriculum.*

Consensus was very high (96.2% positive).

Remark: There were comments on the importance of integrating cough training into respiratory, allergy or ENT specialty training curriculum.

### Statement 16

*Specialty trainees/fellows (*e.g. *respiratory, allergy or ENT) should be required to undertake a period of training/participate in clinics which regularly receive referrals for chronic cough.*

Consensus was very high (92.5% positive).

Remark: There were comments on the importance of training respiratory, allergy or ENT trainees and fellows at cough clinics.

## Discussion

The present Delphi study, based on the NEUROCOUGH CRC network, generated consensus among an international group of clinicians with cough expertise on what the goals and standard procedures for specialist cough clinics should be. Most panellists agreed that specialist cough clinics should be established to provide the optimal care for patients with chronic cough. Consensus was also reached on a minimum panel of diagnostic tests that should be available at cough clinics, comprising chest radiography, spirometry and type 2 inflammatory biomarkers (*F*_ENO_ and blood eosinophils). While recording cough triggers, the use of cough severity rating scales and measuring cough-specific quality of life were considered important in the assessment of patients, there was a lower level of agreement as to the specific tools to use in the clinical setting. Several interesting points were highlighted regarding the roles of specialist cough clinics in promoting clinical research, audit and cough specialty training. These findings provide a framework and should provide direction for those seeking to establish a specialist cough clinic with the additional goals of advancing clinical research and improving the quality of care.

Given the variability in geography, patient epidemiology, referral system or healthcare resource, it would be unrealistic to mandate specific standard procedures across clinics or countries. Therefore, our findings provide the consensus concept of specialist cough clinics, including the broad aims, essential components in standardised care and activity in areas beyond clinical practice. Notably, a very high level of consensus (greater than 90%) was reached in recognising the importance of clinical research (*i.e.* development of registries and providing patients with opportunities to participate in interventional trials of novel cough treatments) and cough specialty training (*i.e.* integrating cough clinic experience into the post-graduate specialty (*e.g.* respiratory, ENT or allergy) training curriculum). To date, cough clinics have been central to the recruitment of well-defined patients with refractory chronic cough to smaller earlier phase clinical trials. However, establishing a global network of specialist clinics will support the conduct of larger later phase studies of new cough treatments. They may also facilitate the generation of real-world data using patient registries and development of clinical evidence that will help clinical decision making in the real world. With development of novel biological therapies for severe asthma, specialist asthma clinics are expanding with better patient care [22, 39, 40]. With the introduction of novel anti-tussive drugs, there should be a change in how we deliver care for patients with chronic cough and specialist cough clinics are expected to play central roles in advancing cough patient care. That said, the statement concerning the establishment of specialist cough clinics in every secondary or tertiary respiratory, allergy or ENT care facility was the only one not to achieve consensus. This may reflect perceived challenges in setting up a service which may overlap with pre-existing models of care or need to compete with other healthcare priorities. Advances in telemedicine may provide opportunities to provide specialist cough clinic care in more healthcare facilities and across low-income countries and geographically isolated regions of the world.

The advantages and requirements of establishing cough clinic services and the need for specialty training was mentioned in the British Thoracic Society cough guidelines published in 2006 [41]. However, to the best of our knowledge, education and training specific to cough management is still lacking in most countries.

There are several limitations to this study. First, there is a risk of selection bias because the findings were based on voluntary responses by the NEUROCOUGH members and clinicians recommended by the National Leads. Thus, the perspectives presented in this survey may not be representative of those in each country or region, but are only valid as the opinions of experts constituting the panel. Further inclusion of other specialty clinicians, such as otolaryngologists and gastroenterologists, and primary practitioners interested in setting up cough clinics may increase the external validity. However, this is the first international Delphi study on the topic, consisting of 57 cough experts and clinicians from 19 countries, and the findings should help to guide further expansion. Second, based on the expert opinions, we presented specific items and panels for cough assessment and routine diagnostic evaluation. We acknowledge that the level of evidence from expert opinions is among the lowest in the hierarchy of evidence [42] and our findings may not represent best practice. However, a Delphi survey may be a suitable opportunity to determine current consensus on a practical issue where it is difficult address with evidence-based medicine, including cough assessment tools and a minimum panel of routine diagnostic tests. Finally, as commented by the panellists, differences in geography, healthcare systems or resources should be taken into consideration when implementing the consensus statements. Digital health technologies might help to improve patient care through virtual cough clinics or remote patient monitoring, although the technology needs to be refined and validated before optimal clinical utility is realised.

In conclusion, based on the international expert consensus, this Delphi study proposed the goals and standard procedures for clinicians currently running specialist cough clinics and those interested in establishing the service. The next step is to develop action plans based on our findings to promote the establishment of specialist cough clinics, ensure high quality of care, and promote clinical research and cough specialty training.

## Supplementary material

10.1183/23120541.00618-2023.Supp1 **Please note:** supplementary material is not edited by the Editorial Office, and is uploaded as it has been supplied by the author.
Supplementary material 00618-2023.SUPPLEMENT
