# Supplementary material for: Consensus goals and standards for specialist cough clinics: the NEUROCOUGH international Delphi study
Source: ERJ Open Res. 2023 Nov 20;9(6):00618-2023. doi: 10.1183/23120541.00618-2023 (PMC10658629; doi:10.1183/23120541.00618-2023)
Supplement: Supplementary file 1 [file 00618-2023.SUPPLEMENT.pdf]

Online Repository

TITLE: Consensus Goals and Standards for Specialist Cough Clinics: the NEUROCOUGH International Delphi Study

Table E1. List of original statement and survey item draft

1. Statement: Specialist cough clinics should be set up to provide the optimal care for patients with cough.
2. Statement: Aims of specialist cough clinics should be to improve patient outcomes, avoid inappropriate investigations and treatments, and advance clinical research and drug development.
3. Statement: Cough clinics should be supervised by clinicians with expertise in cough management.
4. Statement: Cough clinics should provide evaluation and management of patients according to the agreed procedures defined by national and/or international consensus.
5. Statement: Cough clinics should be established in every secondary respiratory or allergy care facility.
6. Statement: In cough clinics, cough should be routinely assessed using validated, or at least structured cough measurement tools.
6-1. Item importance rating: Please rate the importance of each established item as a routine assessment tool in cough clinics.<br>① Cough severity numerical rating scale (e.g., 0-10 score or modified Borg scale)<br>② Cough severity visual analogue scale (e.g., 0-100 mm)<br>③ Cough frequency (ambulatory cough monitoring)<br>④ Cough-specific impact or QoL (e.g., LCQ or CQLQ)<br>⑤ Generic health QoL (e.g., EuroQoL or SF-36)<br>⑥ Airway Reflux Questionnaire (e.g., HARQ)<br>⑦ Cough severity diary
6-2. Item importance rating: Please rate the importance of each item as additional measure that should be routinely used in cough clinics, although the assessment tool

may need development or validation.<br>① Subjective cough frequency<br>② Cough intensity<br>③ Cough triggers<br>④ Cough complications such as tiredness, pain, incontinence, sleep disturbance, work ability, family life<br>⑤ Urge to cough<br>⑥ Throat sensations
7. Statement: In every patient newly referred with chronic cough, a minimum panel of routine tests should be undertaken (or reviewed).
7-1. Item importance rating: Please rate the importance of each item as a routine test to undertake or review in every newly referred patient with chronic cough.<br>① Chest X-ray<br>② Spirometry<br>③ Reversibility test<br>④ FeNO<br>⑤ Blood eosinophils<br>⑥ Sputum eosinophils<br>⑦ Allergy skin test (or serum specific IgE test)<br>⑧ Methacholine challenge test<br>⑨ Mannitol challenge test<br>⑩ Sinus imaging<br>⑪ Nasal endoscopy<br>⑫ Laryngoscopy<br>⑬ 24-hr esophageal pH<br>⑭ High resolution esophageal manometry<br>⑮ GI endoscopy<br>⑯ Cough challenge test<br>⑰ Bronchoscopy
8. Statement: Decision to commence opiates (as anti-tussives) should be carefully made by cough specialist physicians.

9. Statement: Decision to commence current neuromodulators (such as gabapentin or amitriptyline, as anti-tussives) should be carefully made by cough specialist physicians.
10. Statement: In cough clinics, cough control therapy, or speech language and pathology therapy, should be accessible.
11. Statement: In cough clinics, multi-disciplinary team meetings should take place regularly to discuss appropriate patients.
12. Statement: Where possible, cough clinics should provide an opportunity for patients to participate in clinical trials of novel cough therapies.
13. Statement: Cough clinics should participate in local and international audit on an ongoing basis with the aim of providing high-quality cough services.
13-1. Item importance rating: Please rate the importance of each item as a quality indicator for cough clinical service.<br>① Presence of clinicians with expertise in cough management<br>② Adherence to the agreed procedures defined by national and/or international consensus in patient management<br>③ Quantification of baseline cough severity or impact using established tools<br>④ Quantification of treatment response at follow up consultation using established tools<br>⑤ Accessibility to chest X-ray<br>⑥ Accessibility to spirometry<br>⑦ Accessibility to FeNO, blood eosinophils, or sputum eosinophils<br>⑧ Accessibility to allergy skin test (or serum specific IgE test)<br>⑨ Accessibility to methacholine or mannitol challenge test<br>⑩ Accessibility to sinus imaging and nasal endoscopy<br>⑪ Accessibility to laryngoscopy<br>⑫ Accessibility to 24-hr esophageal pH<br>⑬ Accessibility to High resolution esophageal manometry<br>⑭ Accessibility to GI endoscopy<br>⑮ Accessibility to Cough challenge test<br>⑯ Accessibility to Bronchoscopy<br>⑰ Accessibility to Cough control therapy (or speech language and pathology

therapy)<br>⑱ Multidisciplinary team
14. Statement: Cough evaluation and management should be integrated into the post graduate specialty (e.g., respiratory or allergy) training curriculum.
15. Statement: Specialty trainees/fellows (e.g., respiratory or allergy) should be required to undertake a period of training/participate in clinics which regularly receive referrals for chronic cough.

Table E2. List of 57 panelists

Country	Panelist
Australia	Peter Gibson (Department of Respiratory and Sleep Medicine, John Hunter Hospital, Newcastle, NSW, Australia; Priority Research Centre for Healthy Lungs, The University of Newcastle)
Belgium	Charlotte Van de Kerkhove (Respiratory Oncology Unit (Respiratory Diseases), University Hospital KU Leuven)<br>Lieven Dupont (Department of Pneumology, UZ Leuven, Leuven, Belgium; Respiratory Diseases and Thoracic Surgery, Department of Chronic Diseases and Metabolism, KU Leuven)
Canada	Andréanne Côté (Institut de recherche de cardiologie et de pneumologie de Quebec-université Laval)<br>Andrew Thamboo (Division of Otolaryngology- Head & Neck Surgery, University of British Columbia)<br>Diane Lougheed (Department of Medicine, Queen's University Ringgold standard institution)<br>Imran Satia (Department of Medicine, McMaster University)<br>Louis-Philippe Boulet (Quebec Heart and Lung Institute, Laval University)<br>Michael Cyr (Division of Clinical Immunology & Allergy, McMaster University)<br>Parameswaran Nair (Division of Respirology, St Joseph’s Healthcare and McMaster University)<br>Patrick Mitchell (Division of Respirology, Department of Medicine, McMaster University)<br>Stephen K. Field (Division of Respiratory Medicine, Cumming School of Medicine, University of Calgary)<br>Terence Ho (Department of Medicine, McMaster University)
China	Kefang Lai (The First Affiliated Hospital of Guangzhou Medical University)<br>Zhongmin Qiu (Department of Respiratory Medicine, Shanghai Tongji Hospital)
Finland	Anne Lätti (School of Medicine, Faculty of Health Sciences, University of Eastern Finland)<br>Hanna Nurmi (School of Medicine, Faculty of Health Sciences, University of Eastern Finland)<br>Heikki Koskela (School of Medicine, Faculty of Health Sciences, University of Eastern Finland)
France	Laurent Guilleminault (Department of respiratory and allergic diseases, Toulouse University, Hospital Center)
Germany	Ludger Klimek (Center for Rhinology and Allergology, Wiesbaden)<br>Peter Kardos (Centre of Allergy, Respiratory and Sleep Medicine, Maingau Clinic of the Red Cross, Frankfurt am Main)

Israel	Ram Mor (Pulmonary Institute, The Haim Sheba Medical Center)
Italy	Giovanni Fontana (Department of Experimental and Clinical Medicine, University of Florence)
Japan	Akio Niimi (Department of Respiratory Medicine, Allergy and Clinical Immunology, Nagoya City University Graduate School of Medical Sciences)<br>Hisako Matsumoto (Department of Respiratory Medicine & Allergology, Kindai University Faculty of Medicine)
Korea	Byung Jae Lee (Division of Allergy, Department of Medicine, Samsung Medical Center, Sungkyunkwan University School of Medicine)<br>Sang Heon Cho (Division of Allergy and Clinical Immunology, Department of Internal Medicine, Seoul National University College of Medicine)<br>Woo-Jung Song (Asan Medical Center, University of Ulsan College of Medicine)<br>Yoon Seok Chang (Division of Allergy and Clinical Immunology, Department of Internal Medicine, Seoul National University Bundang Hospital, Seoul National University College of Medicine)
Latvia	Madara Tirzīte (Riga Stradins University)
Netherlands	Jan Willem K van den Berg (Isala Hospital)
Poland	Adam Barczyk (Department of Pneumonology, Medical University of Silesia)<br>Ewa Jassem (Department of Pneumonology, Medical University of Gdansk)<br>Marta Dąbrowska (Department of Internal Medicine, Pulmonary Diseases and Allergy, Medical University of Warsaw)
Slovakia	Renata Péčová (Department of Pathophysiology, Jessenius Faculty of Medicine, Comenius University in Bratislava)
Spain	Christian Domingo Ribas (Pulmonary Service, Corporació Sanitària Parc Taulí (Sabadell), Dept of Medicine, Universitat Autònoma de Barcelona)<br>Gonzalez-Barcala Francisco-Javier (Department of Respiratory Medicine, Hospital Clínico Universitario de Santiago de Compostela)
Sweden	Eva Millqvist (Department of Internal Medicine/Respiratory Medicine and Allergology, Sahlgrenska University Hospital, University of Gothenburg)<br>Össur Ingi Emilsson (Department of Medical Sciences, Respiratory, Allergy and Sleep Research, Uppsala University and Department of Respiratory Medicine, Akademiska sjukhuset)
UK	Alice Turner (Institute for Applied Health Research, University of Birmingham)<br>Alyn H Morice (Hull York Medical School, University of Hull, Castle Hill Hospital)<br>Chris Brightling (Institute for Lung Health, Leicester NIHR BRC, University of Leicester)<br>Ian Pavord (Respiratory Medicine Unit and Oxford Respiratory NIHR BRC, Nuffield Department of Clinical Medicine, University of Oxford)<br>Jaclyn A Smith (University of Manchester and Manchester University NHS Foundation

	Trust)<br>James H Hull (Royal Brompton Hospital)<br>Kian Fan Chung (Experimental Studies Unit, National Heart & Lung Institute, Imperial College London)<br>Lorcan P McGarvey (Wellcome-Wolfson Institute for Experimental Medicine, School of Medicine, Dentistry and Biomedical Sciences, Queen's University Belfast)<br>Sean Parker (Northumbria Healthcare NHSFT, North Tyneside General Hospital)<br>Surinder Birring (Centre for Human and Applied Physiological Sciences, School of Basic and Medical Biosciences, Faculty of Life Sciences and Medicine, King's College London)<br>Tim Harrison (University of Nottingham)
US	Alan Goldsobel (Allergy and Asthma Associates of Northern California)<br>Krishna M Sundar (Division of Pulmonary & Critical Care Medicine, University of Utah)<br>Mandel R Sher (Division of Allergy and Immunology, Department of Pediatrics, Morsani College of Medicine, University of South Florida)<br>Peter Dicpinigaitis (Albert Einstein College of Medicine and Montefiore Medical Center Bronx)<br>Rachel Taliercio (Departments of Pulmonary Medicine and Critical Care Medicine, Cleveland Clinic Lerner College of Medicine of Case Western Reserve University)<br>Vivek Iyer (Division of Pulmonary and Critical Care Medicine, Mayo Clinic)<br>William Storms (The William Storms Allergy Clinic)
